# Fixed-bearing vs mobile-bearing prostheses for total knee arthroplasty after approximately 10 years of follow-up: a meta-analysis

**DOI:** 10.1186/s13018-021-02560-w

**Published:** 2021-07-06

**Authors:** Dongsheng Hao, Junjie Wang

**Affiliations:** 1 Shanxi Bethune Hospital, Shanxi Academy of Medical Science, No. 99, Longcheng Street, Taiyuan, 030032 Shanxi Province China; 2 Qingdao University, Qingdao, China

**Keywords:** Fixed-bearing, Mobile-bearing, Total knee arthroplasty, Meta-analysis, Randomized controlled trials

## Abstract

**Background:**

The benefits and risks of fixed-bearing and mobile-bearing designs for total knee arthroplasty (TKA) were compared, and long-term functional, clinical and radiological outcomes were analysed.

**Methods:**

A comprehensive search in the PubMed, EMBASE, Web of Science and Cochrane Central databases was conducted to identify randomized controlled trials (RCTs) comparing fixed-bearing and mobile-bearing designs with no less than 9 years of follow-up. Primary outcome measures were Knee Society Scores (KSSs), range of motion (ROM) in knee joint values, complication rates and revision rates. The final search was performed on 23 April 2021.

**Results:**

Six RCTs were included. A total of 451 patients with 612 knees met the inclusion criteria. The mobile-bearing design, in contrast to the fixed-bearing design, can clearly increase the KSFSs, especially with posterior cruciate retention. There was no significant difference in the KSKSs, ROM values, revision rates or complication rates between the two bearing design groups.

**Conclusion:**

After approximately 10 years of follow-up, the mobile-bearing design has advantages in KSFSs over the fixed-bearing design. The mobile-bearing design may also have advantages in the revision rates over the fixed-bearing design when the posterior cruciate ligament is substituted. There may be no clear difference in KSKSs, ROM values or complication rates between these two designs.

## Introduction

Joint diseases such as osteoarthritis (OA) and rheumatoid arthritis (RA) in the knees can lead to topical pain, loss of joint function and a poor quality of life. If the damage and pain in the knee due to arthritis are too severe, joint replacement surgery is required. Total knee arthroplasty (TKA) is a reliable and prevalent end-stage arthritis orthopaedic operation that can reduce pain and enhance the physical function of the knee. With the occurrence of knee OA and RA continuing to increase, it is expected that the number of TKAs will increase exponentially in the future [1, 2]. However, long-term follow-up data have revealed risks for loosening and wear of the implants often leading to revision surgery. Approximately 10% of TKAs require revision surgery and loosening and wear account for approximately 21% of all revisions [3, 4]. Loosening is related to stresses at the bone fixation site, whereas wear is mainly due to a lack of congruency during implant motion [5]. To prolong the lifespan of implants, TKA has undergone a rapid technological development phase in recent years. The results of this technological development have been applied in clinical practice and more recent TKA designs have sought to increase the congruency without increasing the stress on the implant fixation site [6], thus reducing the risk for loosening and wear.

Fixed-bearing and mobile-bearing are two kinds of bearing designs for TKA. A fixed-bearing knee design has round femoral components that articulate with a relatively flat tibial articular surface. Although this configuration allows for some axial rotation, it results in high contact stress between the femoral and tibial surface. Because of these circumstances, the concept of a mobile-bearing knee design was developed. Due to its motion at the tibia-insert interface, greater tibiofemoral congruency can be achieved, reducing wear on the implants and reproducing more natural kinematics of the knee; these processes are not accompanied by an increase in the stress at the bone-implant interface [7], resulting in increased durability and knee function. Laboratory testing and computer modelling have confirmed that mobile-bearing designs helps to minimize wear by reducing delamination and fatigue fractures in theory [8]. However, the study also suggested that the additional surface wear could offset the reduction in wear at the femorotibial articulation site [9]. To compare the benefits and harms of fixed-bearing versus mobile-bearing TKAs, numerous comparative studies have been conducted in the past two decades, and most studies concluded that there is no difference between fixed-bearing and mobile-bearing designs with regard to pain, range of motion (ROM) or function [10, 11]. A previous meta-analysis also concluded that mobile-bearing designs had similar effects on knee pain, clinical and functional scores, health-related quality of life scores, revision surgery rates, mortality rates, reoperation rates and other serious adverse events compared with fixed-bearing designs. However, they included only studies that evaluated posterior cruciate-retaining TKA, and the follow-up duration of their included studies was not long enough [12]. It is widely accepted that posterior cruciate-retaining TKA can maintain stability from extension to flexion, while posterior cruciate-substituting TKA can increase the flexion and extension gaps [13, 14]. Considering that cruciate retaining can influence knee function, it is necessary to perform a meta-analysis that includes long-term follow-up results and posterior cruciate-substituting TKAs.

This article compares the benefits and harms of fixed-bearing and mobile-bearing TKAs for some long-term functional, clinical and radiological outcomes with a meta-analysis of randomized controlled trials (RCTs).

## Materials and methods

### Search strategy

Two trained researchers independently searched major online databases, including PubMed, EMBASE, the Web of Science and the Cochrane Central Register of Controlled Trials, on 23 April 2021. Different combinations of the following terms were used to perform the search: “total knee arthroplasty”, “total knee replacement”, “knee arthroplasty”, “knee replacement”, “total knee”, “mobile bearing”, “fixed bearing” and “rotating platform”.

### Study identification and eligibility criteria

The two researchers independently screened the titles and abstracts in the online databases and excluded ineligible studies. Subsequently, they read the full texts to include eligible studies that met the following criteria: (1) the participants were individuals who suffered from severe knee diseases; (2) the intervention included TKA surgery that compared fixed-bearing and mobile-bearing designs; (3) the anticipated outcomes included values that evaluate knee function, knee joint ROM, revision rates or other associated values; (4) the follow-up duration was at least 9 years on average; (5) the type of study was an RCT; and (6) the full text was published in English.

### Data extraction and assessment of risk of bias

Two researchers screened the included studies and extracted the following information: first author name, publication year, study population country, number of participants allocated to each group, number of male participants in each group, number of knees in each group, number of diabetic patients, number of alcohol abusers, number of smokers, number of immunosuppressed individuals, mean age of each group, mean body mass density (BMI) of each group, type of TKA (posterior cruciate-retaining or not), implant manufacturer of each group and follow-up duration of each group. Disagreements were resolved with the help of a third investigator. The Cochrane risk of bias tool was used to evaluate the methodological quality of the studies [15].

### Statistical analysis

The two investigators identified and recorded the following outcomes: Knee Society Scores (KSSs), ROM, complication rates and revision rates. The KSSs include the KSKSs, which are mainly used to evaluate pain, ROM, and knee stability, and the KSFSs, which are used to evaluate a patient’s ability to walk and climb stairs [16].

The meta-analysis was performed using RevMan 5.3. Odds ratios (ORs) and 95% confidence intervals (CIs) were calculated for the dichotomous variables, and a random-effects model was used. The mean difference (MD) and 95% CIs were calculated for continuous variables, and a fixed-effects model was used. Statistical significance was deemed to exist when *P* < 0.05. The heterogeneity across studies in comparisons was identified using the chi-square (χ^2^) and I^2^ tests; if *P* < 0.05 and I^2^ > 50%, heterogeneity was considered to exist. For continuous variables, if *P* < 0.05 and I^2^ > 50%, a random-effects model was applied.

## Results

### Literature search

**Fig. 1:**
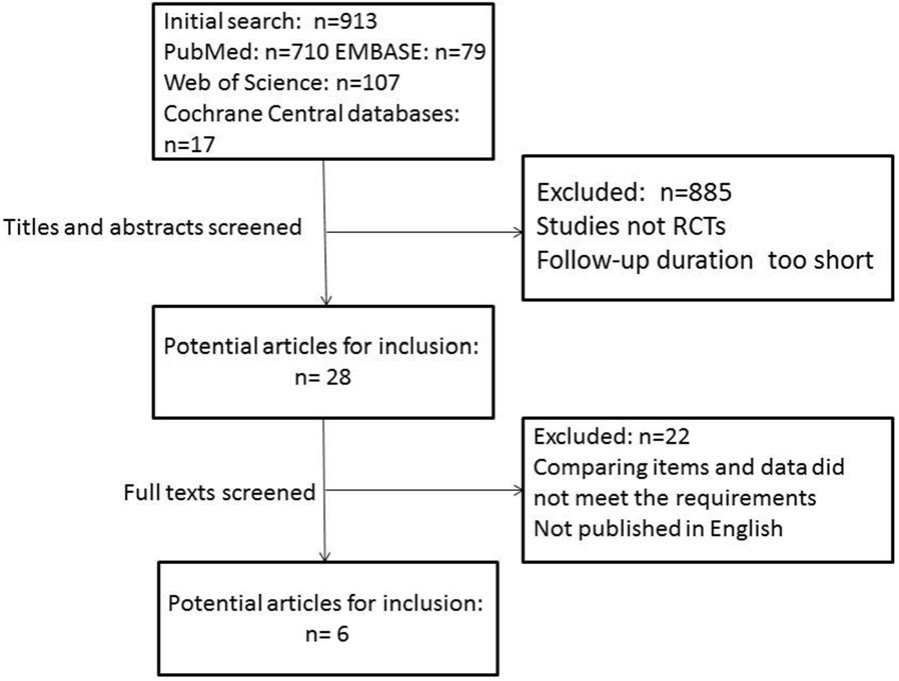
Flow diagram of studies included in this meta-analysis

In total, 913 studies were screened using our search method after duplicates were removed. Subsequently, 885 titles were not eligible for inclusion after the titles and abstracts were read, and 28 trials remained that required a full-text review. Afterwards, 22 articles were eliminated because their full texts were either not published in English or the comparison items did not meet our requirements. Finally, 6 articles were eligible for this meta-analysis [17–22] (Fig. 1).

### Study characteristics

Table 1 provides detailed baseline data for the 6 included studies. The publication year ranged from 2009 to 2018. The sample size of each group ranged from 33 to 122 patients. In total, 451 patients with 612 knees were included. Two studies involved posterior cruciate-retaining TKA, and most studies, with one exception, utilized implants that were manufactured by the same company in both groups [21]. The mean follow-up duration ranged from 9 years to 12 years. None of the included studies reported that they enrolled diabetic patients, alcohol abusers, smokers or immunosuppressed patients.

**Table 1:** Characteristics of the included studies

First author	Young-Hoo Kim	Steven T. Woolson	B. G. Pijls	N. Poirier	M. P. Abdel	A. J. Powell
Publication year	2009	2011	2012	2015	2018	2018
Country	South Korea	USA	Netherlands	France	USA	New Zealand
Number of patients in fixed/mobile group	61/61	26/23	33	31/30	61/53	72
Number of male patients in fixed/mobile group	16	–	5/3	13/14	–	–
Number of knees in fixed/mobile group	122/122	30/31	21/21	31/30	66/53	46/39
Mean age in fixed/mobile group	48.3 (range 34–55)	77.9 (range 56–96)/78 (range 48–91)	66 (SD 14)/64 (SD 11)	72 (SD 6)/70 (SD 6)	–	–
Mean BMI in fixed/mobile group	–	29.2/27.7	27 (SD 5.4)/27 (SD 3.1)	–	–	–
Posterior cruciate-retaining or not	Yes	No	No	No	No	Yes
Manufacturer of implants in fixed/mobile group	DePuy/DePuy	Zimmer/DePuy	Stryker-Howmedica/Stryker-Howmedica	Zimmer/Zimmer	DePuy Synthes/DePuy Synthes	DePuy Synthes/DePuy Synthes
Follow-up duration	Mean 10.8 years (range, 10–12 years)	10 years (range 116–160 months)	10 to 12 years	9 years (SD 1.3)	Median 10 years (IQR 9.2 to 10.4)	10 years

SD, standard deviation; IQR, interquartile range; BMI, body mass index

### Study quality

**Fig. 2:**
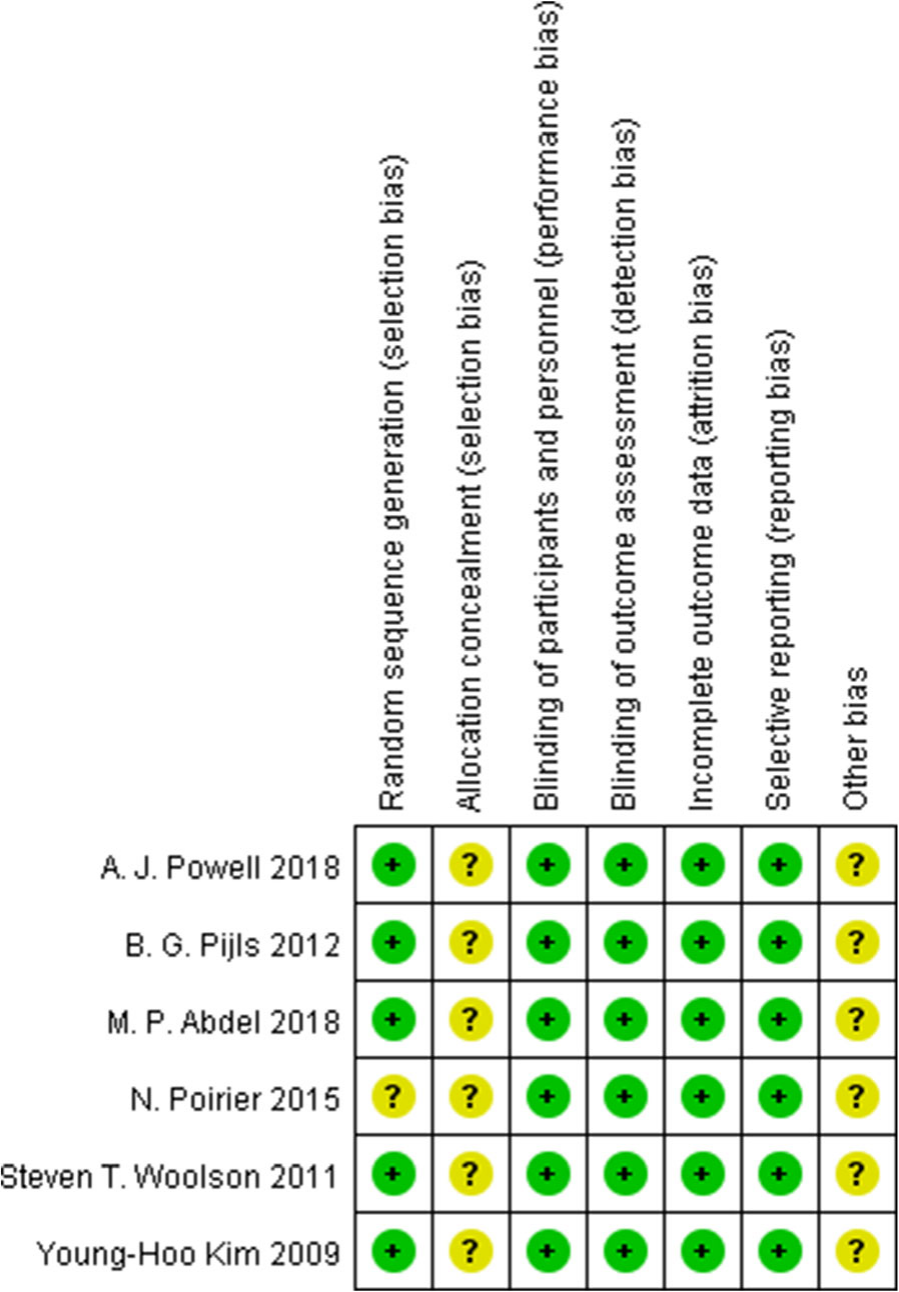
Quality of the included studies

The methodological quality of all RCTs included was deemed to be relatively high (Fig. 2), with most aspects evaluated as having a low risk of bias.

According to Egger et al. [23], using the symmetry of a funnel plot to assess publication bias is not a precise method for a meta-analysis that includes 10 or fewer studies. Therefore, a funnel plot was not presented in this meta-analysis.

### Knee Society Knee Scores (KSKSs)

**Fig. 3:**
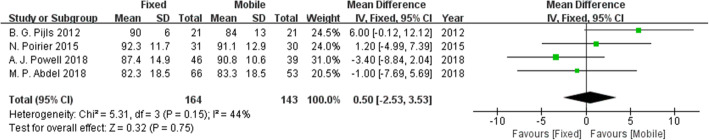
Forest plot for KSKSs. There was no clear difference in the KSKSs between the fixed-bearing design and mobile-bearing design

After screening the 6 studies, we found that KSKSs were reported by more than one study. Figure 3 indicates no clear difference in the KSKSs between the fixed-bearing design and mobile-bearing design.

### Knee Society Function Scores (KSFSs)

**Fig. 4:**
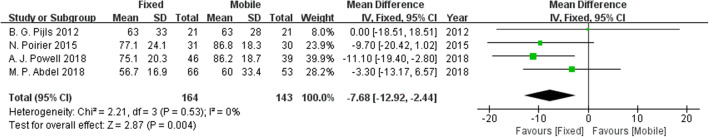
Forest plot for KSFSs. The mobile-bearing design, in contrast to the fixed-bearing design, can significantly increase KSFSs.

KSFSs were reported in four studies. Figure 4 shows that the mobile-bearing design, in contrast to the fixed-bearing design, can significantly increase KSFSs.

### ROM

**Fig. 5:**
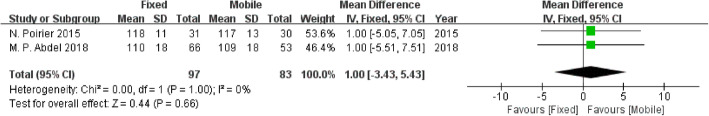
Forest plot for ROM. There was no significant difference between the fixed-bearing and mobile-bearing design for ROM.

Detailed information on ROM was provided in two studies. Figure 5 demonstrates that there was no significant difference between the fixed-bearing and mobile-bearing design for ROM.

### Revision rates

**Fig. 6:**
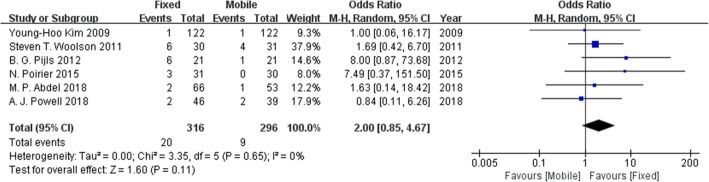
Forest plot for revision rates. No difference was found in the revision rates between the fixed-bearing and mobile-bearing designs.

Six studies investigated the revision rates. Figure 6 suggests that no difference in the revision rates was found between the fixed-bearing and mobile-bearing designs.

### Complication rates

Four complications were reported by more than one study and were included in our meta-analysis: aseptic loosening, deep infection, radiolucent lines and osteolysis. Table 2 presents the results of the pooled statistical analysis and shows that there was no difference in complication rates between the fixed-bearing and mobile-bearing designs.

**Table 2:** The results of the forest plots for complication rates

Complication	Number of patients	Number of included studies	OR	95% CI	P value	χ^2^	I^2^	Effect model
Aseptic loosening	246	3	2.72	0.42, 17.58	0.29	0.99	0%	Random effects
Deep infection	490	4	0.58	0.14, 2.39	0.45	0.67	0%	Random effects
Radiolucent line	329	2	2.10	0.51 ,8.69	0.30	0.24	28%	Random effects
Osteolysis	366	3	1.87	0.21, 16.65	0.58	0.08	61%	Random effects

### Subgroup analyses

**Fig. 7:**
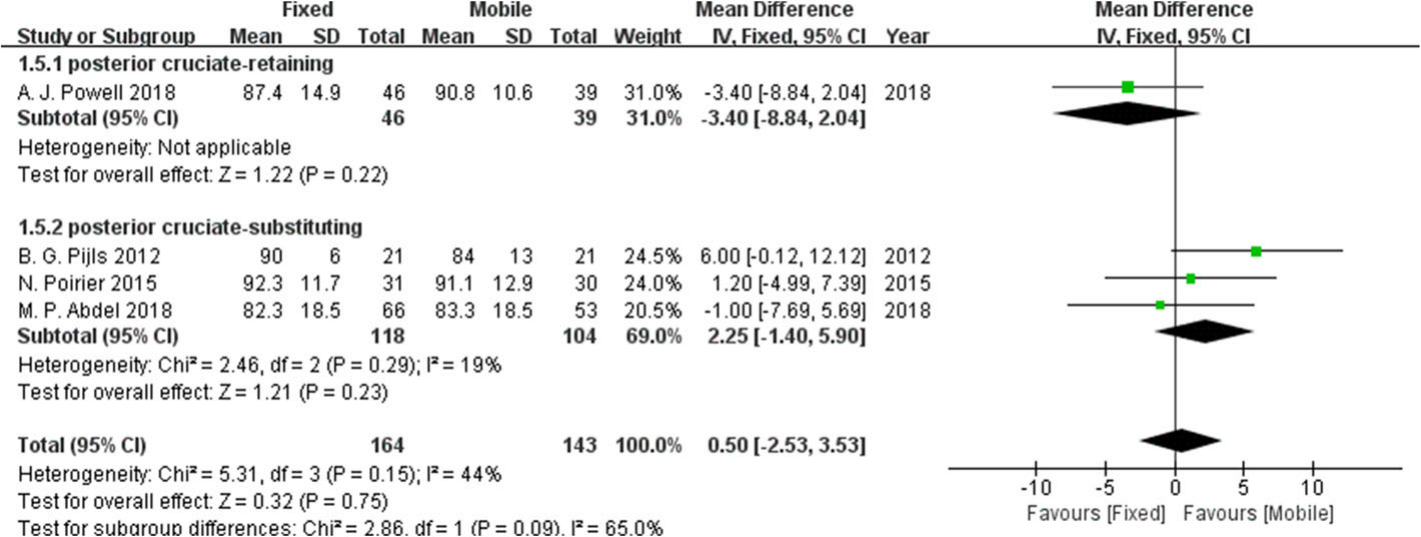
The forest plot of the subgroup analysis of the KSKSs. There was no clear difference in the KSKSs regardless of whether the posterior cruciate ligament was retained

**Fig. 8:**
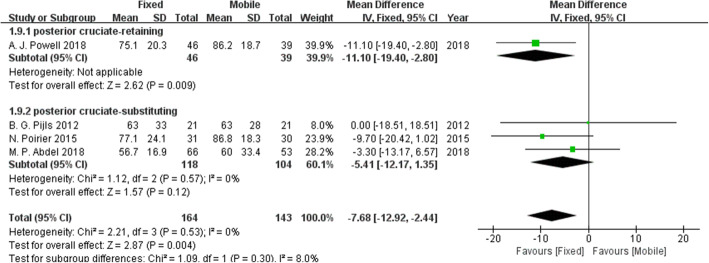
The forest plot of the subgroup analysis of the KSFSs. Compared with the fixed bearing design group, the KSFSs was increased in the mobile-bearing design group with posterior cruciate retention, while there was no significant difference in the KSFSs between the two groups without posterior cruciate retention

**Fig. 9:**
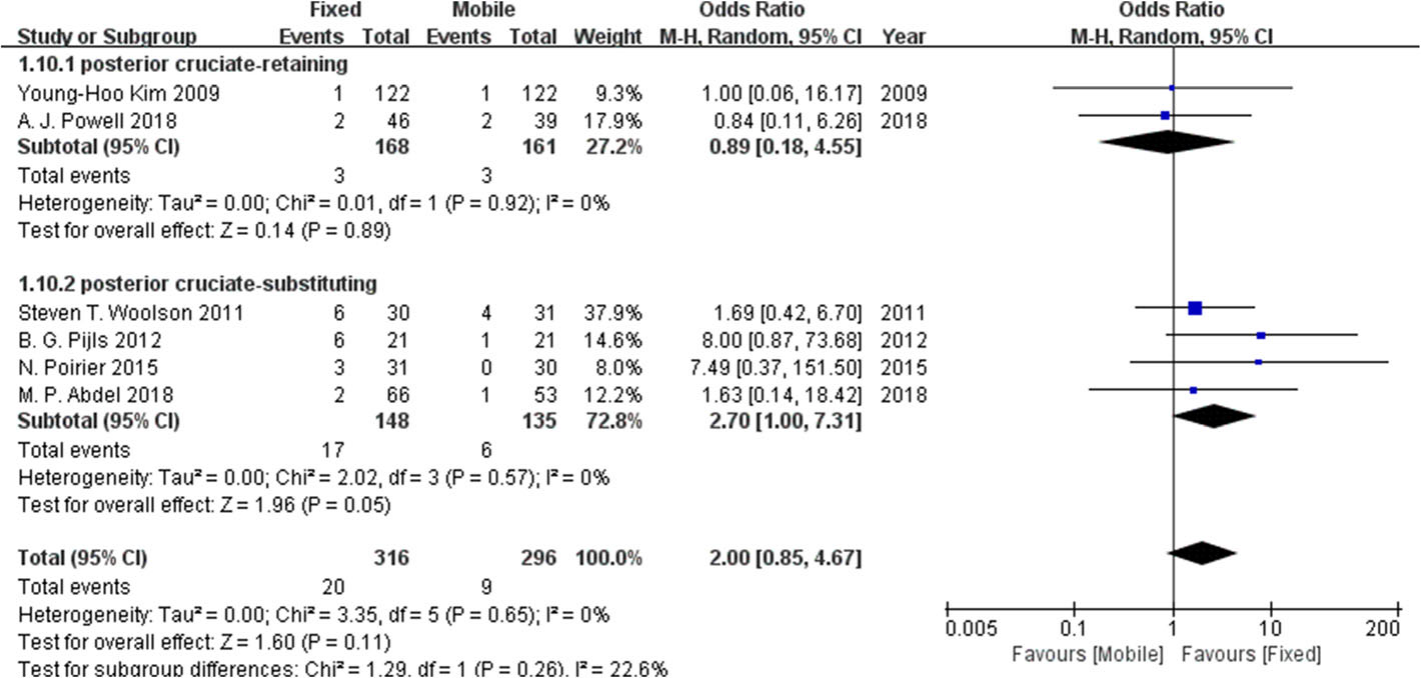
The forest plot of the subgroup analysis of revision rates. There is no clear difference in the revision rates between the fixed-bearing and the mobile-bearing groups regardless of whether the posterior cruciate ligament was retained

**Fig. 10:**
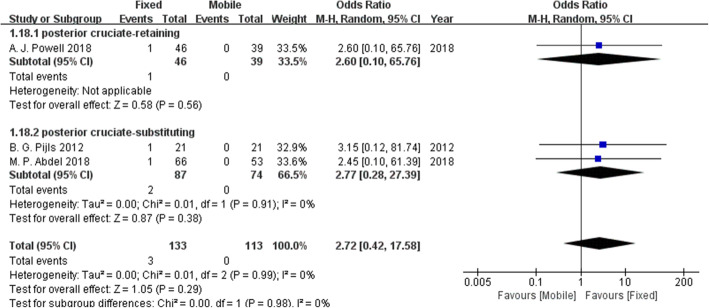
The forest plot of the subgroup analysis of aseptic loosening. There was no clear difference in aseptic loosening between the fixed-bearing and mobile-bearing groups regardless of whether the posterior cruciate ligament was retained

**Fig. 11:**
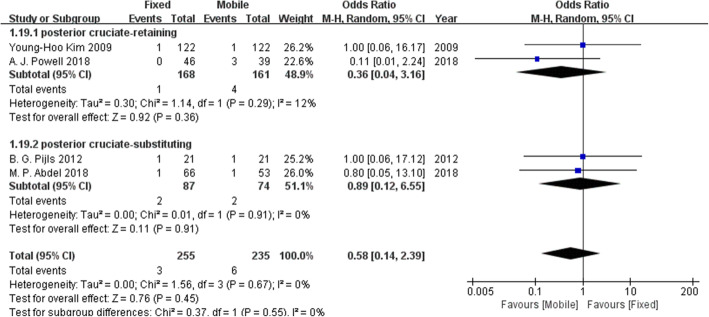
The forest plot of the subgroup analysis of deep infection. There was no clear difference in deep infection between the fixed-bearing and mobile-bearing groups regardless of whether the posterior cruciate ligament was retained

**Fig. 12:**
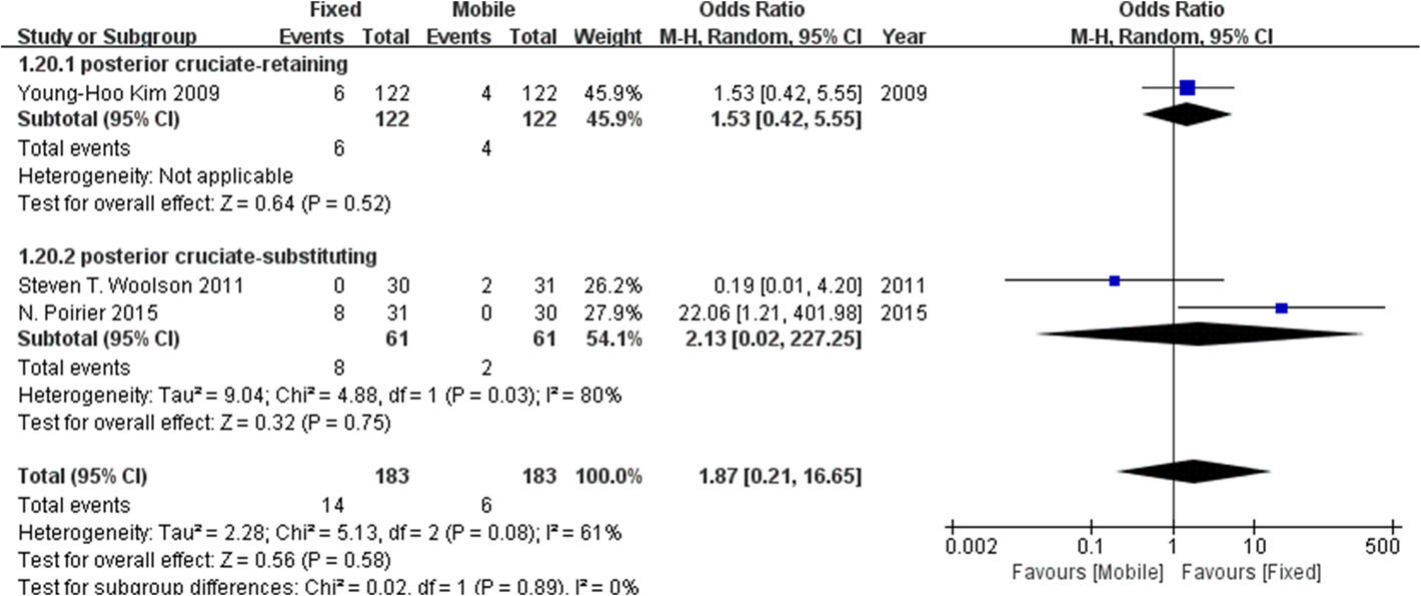
The forest plot of the subgroup analysis of osteolysis. There was no clear difference in osteolysis between the fixed-bearing and mobile-bearing groups regardless of whether the posterior cruciate ligament was retained

To investigate the influence of posterior cruciate-retaining TKA, we conducted subgroup analyses of comparisons with sufficient sample sizes. We established the subgroups based on whether the posterior cruciate ligament was retained. The KSKSs, KSFSs, revision rates, aseptic loosening, deep infection and osteolysis were analysed. Figure 7 shows no clear difference in the KSKSs regardless of whether the posterior cruciate ligament was retained. Figure 8 indicates that compared with the fixed-bearing design, the KSFSs was increased in the mobile-bearing design with posterior cruciate retention, while no significant difference in the KSFSs was found between the two bearing designs without posterior cruciate retention. Figures 9, 10, 11, and 12 show no clear difference in the revision rates, aseptic loosening or deep infection between the fixed-bearing and mobile-bearing designs, regardless of whether the posterior cruciate ligament was retained.

## Discussion

Overall, the forest plot results suggest that the mobile-bearing design, in contrast to the fixed-bearing design, can clearly increase the KSFSs, especially with posterior cruciate retention. There was no significant difference in the KSKSs, ROM values, revision rates or complication rates between the two bearing design groups.

Two types of outcomes were used to compare fixed-bearing and mobile-bearing designs. The KSSs and ROM can be used to assess knee function, while the revision rates and complication rates can be used to evaluate the lifespan of the implants. Our meta-analysis indicated that after approximately 10 years of follow-up, the KSFSs were much higher in the mobile-bearing design group than in the fixed-bearing design group, whereas the difference in KSKSs was not clear between the two designs. This result is quite different from a previous meta-analysis that indicated no significant difference in the KSKSs or KSFSs between the two bearing designs [12]. A possible reason for this discrepancy may be that our follow-up duration was consistently long while the follow-up duration in their study was mixed. In our subgroup analyses, the mobile-bearing design with posterior cruciate retention was associated with an increase in the KSKSs, and the fixed-bearing design with posterior cruciate substitution was associated with an increase in the KSKSs. However, these results may not be considered conclusive because only one study was included in the posterior cruciate retention subgroup, and the p value was not greater than 0.05. Perhaps the small sample size influenced our results. The forest plot showed that the KSFSs were similar between the two subgroups and consistent with the original results. A previous meta-analysis concluded that there were no clinically relevant differences between posterior cruciate-retaining TKA and posterior cruciate-stabilizing TKA in terms of clinical and functional outcomes [24], but their follow-up duration was too short, and the KSSs may have changed over time. Further studies are needed to explore the effect of retention of the posterior cruciate ligament. In addition to the KSSs, ROM can be used to evaluate the knee joint. Our meta-analysis suggests that after approximately 10 years, the fixed-bearing and mobile-bearing designs have nearly identical ROM values, which is consistent with our KSKS results. Further studies are needed to explore the effect of posterior cruciate ligament retention on ROM.

The original aim of the mobile-bearing design was to reduce wear, prolong the lifespan of the implant and reduce the revision rates. Common reasons for revision include aseptic loosening, deep infection, fracture, radiolucent lines, osteolysis, wear and dislocation [3]. Our meta-analysis suggested that the risks for radiolucent lines, osteolysis, aseptic loosening and deep infection were similar for the two bearing designs. In addition, our meta-analysis indicated that there was no clear difference in the revision rates between the two bearing design groups after approximately 10 years; this finding is consistent with previous studies. However, the revision rates in the mobile-bearing group were lower than those in the fixed-bearing group, and our subgroup analysis showed that this tendency in the mobile-bearing group was much stronger in the posterior cruciate-substituting subgroup than in the posterior cruciate-retaining subgroup. The trends for aseptic loosening, deep infection and osteolysis did not change regardless of whether the cruciate was retained. The heterogeneity for osteolysis was relatively large in both the original and subgroup analyses. The small sample size and insufficient follow-up duration may have caused these phenomena. Future studies are needed to acquire more data and confirm our results.

Other values in the included studies compared the benefits and harms of the two bearing designs. For example, B. G. Pijls et al. found that after approximately 10 years of follow-up, there was no significant difference in the femorotibial alignment angle, α angle (frontal angle of the femoral component), β angle (frontal angle of the tibial component) and δ angle (sagittal angle of the tibial component) between the fixed-bearing and mobile-bearing groups [19]. N. Poirier et al. reported that 9 years after surgery, no clear difference was found in subjective patient satisfaction with the surgical outcome between the fixed-bearing and mobile-bearing groups [22], while A. J. Powell et al. indicated that the mobile-bearing group had a significantly higher score on the 12-Item Short-Form Health Survey and Knee Injury and Osteoarthritis Outcome Score assessment than the fixed-bearing group did [20]; however, we speculate that one possible reason may be that their TKA procedure retained the posterior cruciate ligament. In addition, these outcomes were reported in only one study and could not be included in our meta-analysis; further studies are required to explore these variables.

Mobile-bearing TKA was developed to reduce polyethylene contact stress by decreasing the increased wear associated with the fixed-bearing design, thus improving joint function and reducing the revision rates. However, these advantages used to be regarded as theoretical since several clinical trials suggested that the mobile-bearing design had no clear advantage over the fixed-bearing design [25, 26]. In addition, a recent meta-analysis concluded that there was no significant difference in radiostereographic migration rates between mobile-bearing and fixed-bearing implants [27], indicating that the revision rates between the two bearing designs may have no clear difference. Our meta-analysis further compared the benefits and harms between the two bearing designs over approximately 10 years of follow-up and the results showed that the mobile-bearing design had advantages over the fixed-bearing design in KSFSs. The mobile-bearing design may also have advantages in the revision rates. Our study also suggests that posterior cruciate ligament retention may influence the effect of the two designs on KSKSs and revision rates. A previous study showed that KSSs and ROM differ between posterior cruciate ligament retention TKA and posterior cruciate ligament-substituting TKA [28]. Further studies are still needed to explore this field, acquire more data and update our results. In addition, approximately 10 years of follow-up may be insufficient considering the average implant lifespan, and longer trials should be implemented to obtain more long-term data.

Although the methodological quality of nearly all included studies was relatively high, there were some limitations of this meta-analysis. First, the number of included studies was relatively small, which may have affected the results of the forest plots. Additionally, due to the small sample size, we were not able to test for publication bias via funnel plots. Second, only two studies retained the posterior cruciate ligament, and only one of them reported knee function, which may have influenced our results. Third, one study used fixed- and mobile-bearing implants manufactured by different companies, which may have influenced our results. Finally, we analysed only articles published in English, which could have been a source of bias.

## Conclusion

Our meta-analysis of RCTs showed that after approximately 10 years of follow-up, the mobile-bearing design has advantages in KSFSs over the fixed-bearing design. The mobile-bearing design may also have advantages in the revision rates over the fixed-bearing design when the posterior cruciate ligament is substituted. There may be no clear difference in KSKSs, ROM values or complication rates between these two designs. However, the small sample size may have influenced our results, and future studies with longer follow-up durations are needed to obtain additional data and more reliable results in this ambiguous field of research.

## Data Availability

As a meta-analysis, there are no patient data sets.

## Abbreviations

OA: Osteoarthritis
RA: Rheumatoid arthritis
TKA: Total knee arthroplasty
RCTs: Randomized controlled trials
KSSs: Knee Society Scores (KSSs)
ROM: Range of motion
OR: Odds ratio
CI: Confidence interval
SD: Standard deviation
IQR: Interquartile range
BMI: Body mass index
KSKSs: Knee Society Knee Scores
KSFSs: Knee Society Function Scores
